# Indications of Laparoscopic Repeat Liver Resection for Recurrent Hepatocellular Carcinoma

**DOI:** 10.1002/ags3.12493

**Published:** 2021-08-04

**Authors:** Masahiko Kinoshita, Akishige Kanazawa, Shogo Tanaka, Shigekazu Takemura, Ryosuke Amano, Kenjiro Kimura, Hiroji Shinkawa, Go Ohira, Kohei Nishio, Shoji Kubo

**Affiliations:** ^1^ Department of Hepato‐Biliary‐Pancreatic Surgery Osaka City University Graduate School of Medicine Osaka Japan; ^2^ Department of Hepato‐Biliary‐Pancreatic Surgery Osaka City General Hospital Osaka Japan

**Keywords:** difficulty scoring system, laparoscopic repeat liver resection, recurrent hepatocellular carcinoma

## Abstract

**Aim:**

This study aimed to evaluate the indications of laparoscopic repeat liver resection (LRLR) for recurrent hepatocellular carcinoma from the viewpoint of its difficulty.

**Methods:**

One hundred and one patients who underwent LRLR and 59 patients who underwent open repeat liver resection (ORLR) were included. The difficulty was classified according to the preoperative predictive factors for difficult LRLR, including an open approach during previous liver resection, history of two or more previous liver resections, history of previous major liver resection, tumor near the resected site of the previous liver resection, and intermediate or high difficulty with the difficulty scoring system. We compared the surgical outcomes between the LRLR and ORLR groups based on the difficulty class (low‐ or intermediate difficiulty class, 0 to 3 predictive factors; high difficiulty class, 4 or 5 factors).

**Results:**

In the low‐ or intermediate difficiulty class, intraoperative blood loss and the proportion of patients with postoperative complications were significantly lower in LRLR than in ORLR, and the duration of the postoperative hospital stay was significantly shorter in LRLR than in ORLR. In the high difficiulty class, total operative time and operative time before starting hepatic parenchymal resection were significantly longer in LRLR than in ORLR, and there were no significant differences in other surgical outcomes between the two groups.

**Conclusion:**

LRLR is recommended for patients in the low or intermediate difficulty class. However, LRLR does not have an advantage with longer operative time for patients in the high difficulty class compared with ORLR.

## Graphical abstract

Laparoscopic repeat liver resection (LRLR) is recommended for patients in our suggested low or intermediate difficulty class. On the other hands, LRLR does not have an advantage with longer operative time for patients in the high difficulty class, compared with open repeat liver resection.

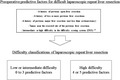

## 1 INTRODUCTION

Liver resection is commonly accepted as a curative treatment for hepatocellular carcinoma (HCC), which is usually featured in treatment guidelines.^1^, ^2^ Although repeat liver resection has also been accepted as an effective treatment for recurrent HCC (HCCR) in patients with preserved liver function,^3^, ^4^, ^5^, ^6^ it is a challenging clinical procedure because the adhesion around the previous liver resection site can often make operative procedures difficult and sometimes results in incidental complications.^7^, ^8^

Laparoscopic liver resection (LLR) is a minimally invasive treatment modality and has demonstrated feasible short‐ and long‐term outcomes comparable to open liver resection.^9^, ^10^, ^11^, ^12^ Recently, the difficulty scoring system (DSS) for initial LLR has been proposed based on the experience at three high‐volume centers in Japan.^13^ A multicenter validation study demonstrated that the DSS could predict the difficulty of surgical procedures and short‐term outcomes in patients who underwent initial LLR.^14^ Although most previous studies have demonstrated the safety of laparoscopic repeat liver resection (LRLR) for patients comparable to initial LLR or open repeat liver resection (ORLR),^15^, ^16^, ^17^, ^18^, ^19^, ^20^, ^21^ it is difficult to compare the advantages and disadvantages of LRLR because the difficulty of LRLR is different from those of initial LLR and ORLR. Therefore, the feasible indication of LRLR remains unclear.

Some previous studies have shown that unfavorable surgical outcomes, such as a long operative time and large intraoperative blood loss, after ORLR or LRLR were associated with a history of previous open liver resection, two or more previous liver resections, the relationship between current and previous locations of liver tumor, and a high DSS score.^15^, ^22^, ^23^, ^24^, ^25^ Our previous study revealed five preoperative predictive factors for difficult LRLR: a history of an open approach during previous liver resection, history of two or more previous liver resections, history of previous major liver resection, tumor near the resected site of the previous liver resection, and intermediate or high difficulty in the DSS.^26^ These are similar to the risk factors for unfavorable surgical outcomes as described above.

This study aimed to evaluate the indications of LRLR by comparing intra‐ and postoperative outcomes of LRLR with those of ORLR, based on risk factors for unfavorable surgical outcomes, including the difficulty classification of LRLR reported in our previous study.

## 2 METHODS

### 2.1 Study population

A total of 244 patients underwent repeat liver resection for HCCR at the Departments of Hepato‐Biliary‐Pancreatic Surgery, Osaka City University Hospital and Osaka City General Hospital between 2010 and 2019. To eliminate operative bias, the patients who underwent repeat liver resection that involved segmentectomy or extensive surgery, noncurative resection, concomitant resection of other organs (except for the gallbladder), resection of multiple lesions, and resection of the caudate lobe (segment 1) were excluded from this study. A total of 160 patients who underwent repeat partial liver resection were included in the study (101 underwent LRLR and 59 underwent ORLR). The median age of the subjects was 70 (range, 32–87) years, and 134 subjects were male. Of the 160 patients, 76 were seropositive for anti‐hepatitis C virus antibody and 42 were seropositive for hepatitis B surface antigen. Eleven patients had alcoholic hepatitis, and five had nonalcoholic steatohepatitis.

This study conformed to the ethical guidelines of the Declaration of Helsinki and was retrospective in nature, and we obtained approval from the Ethics Committees of Osaka City University (No. 3166) and Osaka City General Hospital (No. 1910076). All participants provided written informed consent.

### 2.2 Preoperative risk (predictive) factors for unfavorable surgical outcomes and difficulty classification for LRLR

The preoperative risk factors for unfavorable surgical outcomes after LRLR were selected based on previous studies^15^, ^22^, ^23^, ^24^, ^25^ and our own experiences, including our previous study.^26^ The risk factors in the previous studies^15^, ^22^, ^23^, ^24^, ^25^ included a history of previous open liver resection, two or more previous liver resections, relationship between current and previous locations of liver tumor, and a high DSS score. Our previous study^26^ showed that a history of an open liver resection, history of two or more previous liver resections, history of previous major liver resection (not less than sectionectomy), tumor near the resected site of the previous liver resection (a tumor was classified if it was in the same segment as the previous liver resection site or in an adjacent segment), and intermediate or high difficulty in the DSS were preoperative predictive factors for difficult LRLR. Therefore, the suggested preoperative predictive factors for difficult LRLR^26^ overlapped with the previous reported risk factors for unfavorable surgical outcomes of repeat liver resection.^15^, ^22^, ^23^, ^24^, ^25^ Similarly in this study, in patients who underwent LRLR, all five preoperative predictive factors (history of previous open liver resection, history of two or more liver resections, history of previous major liver resection, tumor near the resected site of the previous liver resection, and intermediate or high difficulty in the DSS) were indicated as independent risk factors for prolonged operative time and/or severe adhesion (Table 1). Prolonged operative time had been defined as >321 min (equivalent to the 75th percentile for the study population; n = 25). Severe adhesion had been defined by the occurrence of one or more of three situations: the patient required >120 min before the start of liver dissection; injury occurred to other organs due to the dissection procedure; or the patient required conversion to open surgery because of the adhesion. Accordingly, 27 patients were classified as having severe adhesion.^26^

**TABLE 1:** Associations between five predictive factors for LRLR and surgical outcomes in patients who underwent LRLR

Variables	Prolonged operative time					Severe adhesion				
	Univariate analysis			Multivariate analysis		Univariate analysis			Multivariate analysis	
	n (%)	*P*‐value	OR	95% CI	*P*‐value	n (%)	*P*‐value	OR	95% CI	*P*‐value
Approach of previous liver resection										
Laparoscopic (n = 52)	7 (13)					6 (12)				
Open (n = 49)	18 (37)	.0061	5.2	1.69‐18.1	.0035	21 (43)	.0003	5.8	1.90‐19.9	.0016
Number of previous liver resections										
One (n = 78)	15 (19)					18 (23)				
Two or more (n = 23)	10 (43)	.018	4	1.15‐15.3	.029	9 (39)	.14			
Tumor near the resected surface of previous liver resection										
No (n = 42)	4 (9.5)					5 (12)				
Yes (n = 59)	21 (36)	.0044	2.3	0.625‐9.75	.21	22 (37)	.0032	4.1	1.14‐17.8	.03
Range of previous liver resection										
less than sectionectomy (n = 81)	18 (22)					18 (22)				
not less than sectionectomy (n = 20)	7 (35)	.26				9 (45)	.047	5.6	1.35‐27.6	.017
Difficulty score^a^										
Low (n = 58)	6 (10)					9 (16)				
Intermediate or high (n = 43)	19 (44)	<.0001	8.9	2.57‐37.8	.0003	18 (42)	.0031	5.5	1.62‐22.9	.0055

Abbreviations: 95% CI, 95% confidence interval; LRLR, laparoscopic repeat liver resection; OR, odds ratio.

^a^ According to the difficulty scoring system.^13^

**FIGURE 1:**
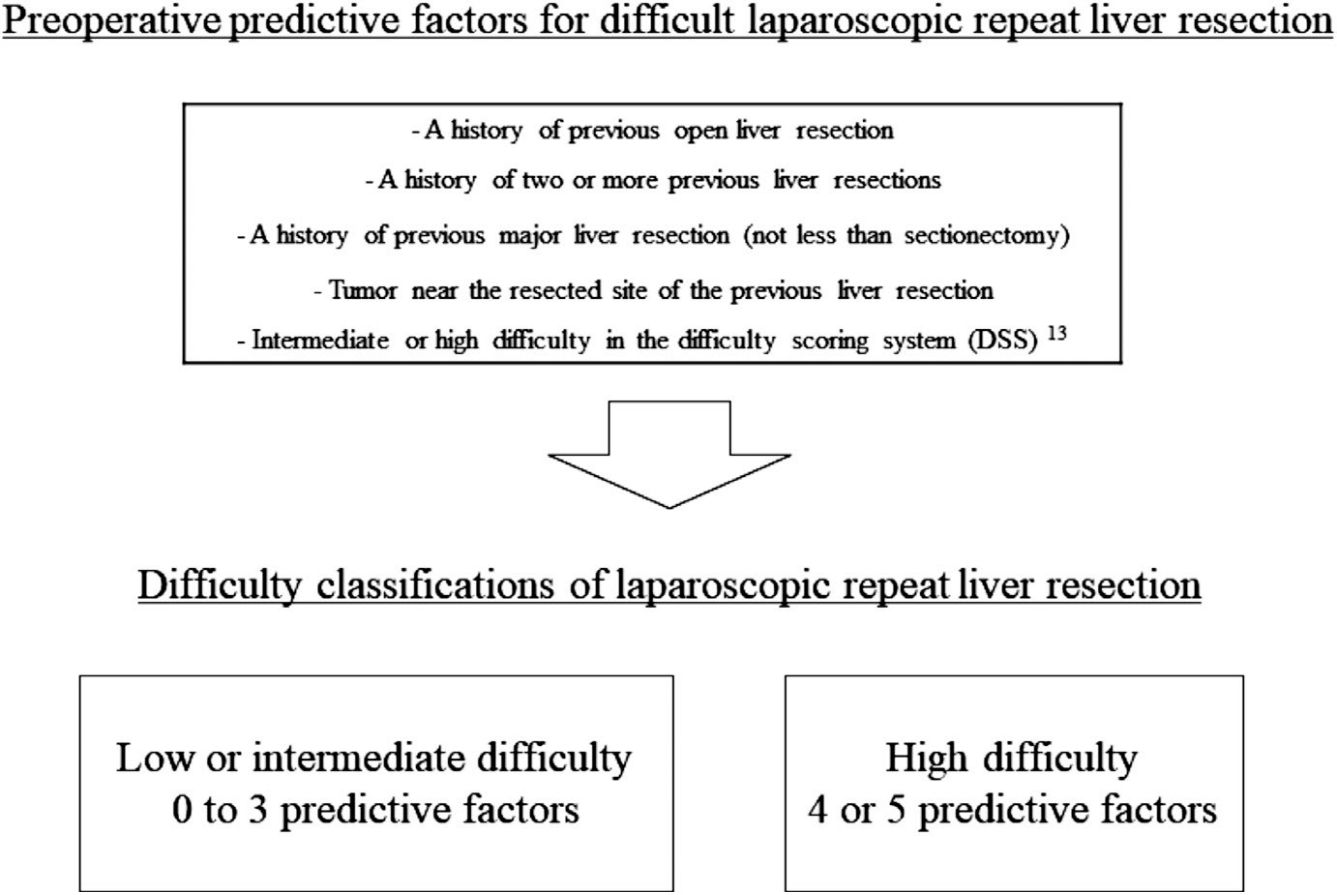
Preoperative predictive factors for difficult laparoscopic repeat liver resection and difficulty classifications suggested in our previous study^26^

In our previous study,^26^ we categorized patients who underwent LRLR into three classifications (patients who had 0 or 1 predictive factor were categorized as having low difficulty, those with 2 or 3 predictive factors as having intermediate difficulty, and those with 4 or 5 predictive factors as having high difficulty) (Figure 1). In the investigation of the feasible indication of LRLR, we compared the surgical outcomes between the LRLR and ORLR groups based on our difficulty classification (low or intermediate difficulty class, 0 to 3 preoperative predictive factors; high difficulty class, 4 or 5 preoperative predictive factors) to avoid bias of surgical difficulty (Figure 1).

### 2.3 Statistical analysis

The Mann–Whitney *U‐*test was used to compare continuous variables. Categorical variables were summarized as numbers and percentages and compared between groups using Fisher's exact test or the chi‐squared test, as appropriate. *P* < .05 was considered statistically significant. Statistical analyses were performed using JMP v. 11 (SAS Institute, Cary, NC).

## 3 RESULTS

### 3.1 **Surgical outcomes in patients who underwent LRLR according to** difficulty **classification**

Surgical outcomes of each difficulty class (low or intermediate and high difficulty class) after LRLR are described in Table 2. In patients who underwent LRLR, significant differences were observed for operative time and intraoperative blood loss and operative time before starting hepatic parenchymal resection between high and other difficulty groups. Rates of conversion to open surgery from LRLR tended to increase in patients with a high difficulty class without a significant difference. Moreover, although the duration of the postoperative hospital stay (in days) did not significantly differ, the incidence of postoperative complications (Clavien–Dindo classification ≥grade IIIa^27^) was significantly higher in high difficiulty groups than in low‐ or intermediate difficiulty groups.

**TABLE 2:** Surgical outcomes in patients who underwent LRLR according to difficulty classification

The number of predictive factors	0 to 3	4 or 5	*P*‐value
Category of difficulty classification	Low or Intermediate (n = 92)	High (n = 9)	
Operative results, median (range)			
Operative time, min	214 (69–704)	425 (153–681)	.0003
Operative time before starting hepatic parenchymal dissection, min	78 (16–410)	223 (30–321)	.0004
Blood loss, mL	50 (2–1500)	330 (3–2170)	.0049
Conversion, n (%)	4 (4.4)	2 (22)	.088
Postoperative outcomes			
Postoperative complication^a^, n (%)	1 (1.1)	3 (33)	.0019
Postoperative hospital stays, median (range), d	8 (2–30)	8 (5–95)	.40

Abbreviation: LRLR, laparoscopic repeat liver resection.

^a^ Clavien–Dindo classification ≥grade IIIa.^27^

### 3.2 Patients’ backgrounds between patients who underwent LRLR and ORLR

Patients’ backgrounds are described in Table 3. There were no differences in age, sex, body mass index, comorbid liver diseases, or tumor diameter between the LRLR and ORLR groups. The proportion of patients with a history of previous open liver resection was significantly lower in the LRLR group than in the ORLR group. The proportion of patients with a history of previous major liver resection tended to be lower in the LRLR group than in the ORLR group. Although there was no difference in the proportion of patients in the low‐ or intermediate difficiulty class between the two groups, the proportion of patients in the high difficiulty class was significantly higher in the ORLR group than in the LRLR group. Comparisons of patient's backgrounds in each difficulty class between patients who underwent LRLR and ORLR are summarized in Tables S1 and S2. In low‐ or intermediate difficiulty class, the proportion of patients with a history of previous open liver resection was significantly lower in the LRLR group than in the ORLR group. Conversely, in the high difficiulty class, the proportion of patients with a history of two or more previous liver resections was significantly higher in the LRLR group than in the ORLR group.

**TABLE 3:** Comparison of preoperative backgrounds between patients who underwent laparoscopic and open repeat liver resection

Variables	LRLR (n = 101)	ORLR (n = 59)	*P*‐value
Age, median (range), years	71 (32–86)	68 (43–87)	.11
Sex, male/female	84/17	50/9	.99
Body mass index, median (range), kg/m^2^	23 (17–39)	24 (17–31)	.69
Comorbid liver disease, n (%)			
Anti‐HCV positive	46 (46)	30 (51)	.62
HBs antigen positive	29 (29)	13 (22)	.46
Alcoholic hepatitis	8 (7.9)	3 (5.1)	.75
Nonalcoholic steatohepatitis	2 (2.0)	3 (5.1)	.36
Tumor diameter, median (range), cm	1.5 (0.4–3.8)	1.7 (0.6–8.5)	.098
A history of previous open liver resection, n (%)	49 (49)	51 (86)	<.0001
A history of two or more previous liver resections, n (%)	23 (23)	14 (24)	0.99
A history of previous major liver resection (not less than sectionectomy), n (%)	20 (20)	19 (32)	.088
Tumor near the resected site of the previous liver resection, n (%)	59 (58)	39 (66)	.40
Intermediate or high difficulty in the difficulty scoring system^a^, n (%)	43 (43)	32 (54)	0.19
Low or intermediate difficulty class^b^, n (%)	92 (91)	44 (75)	.32
High difficulty class^b^, n (%)	9 (8.9)	15 (25)	.0062

Abbreviations: HBs, hepatitis B surface; HCV, hepatitis C virus; LRLR, laparoscopic repeat liver resection; ORLR, open repeat liver resection.

^a^ According to the difficulty scoring system.^13^

^b^ According to our previous study.^26^

### 3.3 **Surgical outcomes between patients who underwent LRLR and** ORLR

A significantly longer total operative time, less intraoperative blood loss, and shorter postoperative hospital stay were observed in the LRLR group than in the ORLR group. There was no difference in the operative time before starting liver parenchymal resection and the proportion of patients with postoperative complications between the groups (Table 4).

**TABLE 4:** Comparisons of surgical outcomes between patients who underwent laparoscopic and open repeat liver resection

Variables	LRLR (n = 101)	ORLR (n = 59)	*P*‐value
Total operative time, median (range), min	221 (69–704)	182 (87–559)	.025
Operative time before starting hepatic parenchymal dissection, median (range), min	80 (16–410)	72 (30–185)	.40
Intraoperative blood loss, median (range), mL	50 (2–2170)	190 (10–1870)	<.0001
Postoperative complications, n (%)^a^	4 (4.0)	7 (12)	.10
Postoperative hospital stay, median (range), d	8 (2–95)	10 (7–50)	.0003

Abbreviations: LRLR, laparoscopic repeat liver resection; ORLR, open repeat liver resection.

^a^ Clavien–Dindo classification ≥grade IIIa.^27^

### 3.4 Comparisons of surgical outcomes between LRLR and ORLR in the low or intermediate difficulty class

In the low or intermediate difficulty class (patients with 0 to 3 preoperative predictive factors), there were no significant differences in the total operative time and operative time before starting hepatic parenchymal resection (Table 5). Intraoperative blood loss was significantly less in the LRLR group than in the ORLR group (*P* < .0001), and the duration of postoperative hospital stay was significantly shorter in the LRLR group than in the ORLR group (*P* < .0001). Moreover, the proportion of patients with postoperative complications was also significantly lower in the LRLR group than in the ORLR group (*P* = .014). In the LRLR group, one patient had bile leakage. In the ORLR group, two patients had bile leakage, one had intractable ascites, and two had pleural effusion.

**TABLE 5:** Comparisons of surgical outcomes between laparoscopic and open repeat liver resection in patients in the low or intermediate difficulty class

Variables	LRLR (n = 92)	ORLR (n = 44)	*P*‐value
Operative results, median (range)			
Total operative time, min	214 (69–704)	185 (87–559)	.16
Operative time before starting hepatic parenchymal dissection, min	78 (16–410)	72 (30–185)	.77
Blood loss, mL	50 (2–1500)	155 (10–1675)	<.0001
Postoperative outcomes			
Postoperative complication^a^, n (%)	1 (1.1)	5 (11)	.014
Postoperative hospital stay, median (range), d	8 (2–30)	10 (7–37)	<.0001

Abbreviations: LRLR, laparoscopic repeat liver resection; ORLR, open repeat liver resection.

^a^Clavien–Dindo classification ≥grade IIIa.^27^

### 3.5 **Comparisons of surgical outcomes between LRLR and ORLR** in **the high difficulty class**

In the high difficulty class (patients with 4 or 5 preoperative predictive factors), total operative time and operative time before starting hepatic parenchymal resection were significantly longer in the LRLR group than in the ORLR group (*P* = .0009 and *P* = .0017, respectively; Table 6). There were no significant differences in intraoperative blood loss and duration of postoperative hospital stay between the two groups. The proportion of patients with postoperative complications was not different between the two groups. In the LRLR group, one patient had bile leakage and two had intractable ascites. In the ORLR group, one patient had bile leakage, one had intractable ascites, and one had pleural effusion.

**TABLE 6:** Comparisons of surgical outcomes between laparoscopic and open repeat liver resection in patients in the high difficulty class

Variables	LRLR (n = 9)	ORLR (n = 15)	*P*‐value
Operative results, median (range)			
Total operative time, min	425 (153–681)	176 (134–295)	.0009
Operative time before starting hepatic parenchymal dissection, min	223 (30–321)	76 (41–131)	.0017
Blood loss, mL	330 (3–2170)	250 (10–1870)	.81
Postoperative outcomes			
Postoperative complication^a^, n (%)	3 (33)	2 (13)	.33
Postoperative hospital stay, median (range), d	8 (5–95)	11 (7–50)	.28

Abbreviations: LRLR, laparoscopic repeat liver resection; ORLR, open repeat liver resection.

^a^Clavien–Dindo classification ≥grade IIIa.^27^

## 4 DISCUSSION

Some previous studies reported that LRLR was not inferior to ORLR in terms of short‐ and/or long‐term outcomes.^15^, ^16^, ^17^, ^18^, ^19^, ^20^, ^21^ They reported less blood loss and a shorter hospital stay in LRLR than in ORLR due to minimal damage to structures surrounding the liver, reduction of adhesion formation, and the need for adhesiolysis in LRLR.^15^, ^16^, ^17^, ^18^ Although these studies concluded that LRLR can be performed safely in selected patients, the definition of such “selected patients” remains unclear. LRLR can often be difficult, and a safe laparoscopic procedure is not always performed for all patients with HCCR. Therefore, in this study we evaluated the surgical outcomes of LRLR and ORLR based on the difficulty of repeat liver resection, based on previous studies^15^, ^22^, ^23^, ^24^, ^25^ and our previous report,^26^ to determine a feasible indication for LRLR.

This study showed that LRLR could be performed with less intraoperative blood loss, and a shorter postoperative hospital stay than ORLR, when all subjects are compared, as previously reported.^15^, ^16^, ^17^, ^18^, ^19^, ^20^, ^21^ In the low or intermediate difficulty class, the intraoperative blood loss and proportion of patients with postoperative complications were significantly lower in the LRLR group than in the ORLR group, and the duration of the postoperative hospital stay was significantly shorter in the LRLR group than in the ORLR group. Therefore, LRLR for HCCR, as a minimally invasive treatment modality, was useful in patients in the low or intermediate difficulty class compared with ORLR. However, in the high difficulty class the total operative time and operative time before starting hepatic parenchymal resection were significantly longer in the LRLR group than in the ORLR group, and there was no statistical superiority in other surgical outcomes in patients who underwent LRLR compared to ORLR. In addition, a high conversion rate (22%) was observed in patients who underwent LRLR in the high difficulty class. The results suggested that LRLR is recommended for patients in the low or intermediate difficulty class (0 to 3 preoperative predictive factors, those with a history of previous open liver resection, history of two or more previous liver resections, history of previous major liver resection, tumor near the resected site of the previous liver resection, and intermediate or high difficulty in the DSS). In contrast, presently LRLR cannot be currently recommended for patients in the high difficulty class (4 or 5 preoperative predictive factors) because LRLR does not have an advantage for such patients, compared with ORLR. In patients in the high difficulty class, LRLR should be recommended to be performed only by an experienced team in high‐volume centers with consideration of conversion to open surgery.

This study included some limitations. First, this was a dual‐center retrospective study and included a small number of patients with some selection biases. Patients who underwent repeat anatomical liver resection or partial resection in the caudate lobe were excluded because there were only a few patients who underwent such procedures laparoscopically. A multicenter study with a large number of patients as a validation study must be performed to assess the more appropriate indications of LRLR for HCCR. Second, recently devices and techniques of laparoscopic surgery are advancing; therefore, our “current” difficulty classification will not be suitable in the future. However, presently the indication of a surgical approach for repeat liver resection is unclear, and our present classifications may be useful to decide the surgical approach in patients who require repeat liver resection. Third, our present study classified the difficulty simply based on the number of predictive factors, with consideration that the five predictive factors were indicated as the independent risk factors in our present and previous studies.^26^ The importance and weight may be different in each factor. A scoring system should be established by a large‐number study, considering the importance and weight of each factor, based on the present results.

In conclusion, the difficulty classification evaluated by five preoperative predictive factors consisting of history of previous open liver resection, history of two or more previous liver resections, history of previous major liver resection (not less than sectionectomy), tumor near the resected site of the previous liver resection, and intermediate or high difficulty in the DSS was useful in making the decision for the indication of LRLR. LRLR is recommended for patients in the low or intermediate difficulty class (0 to 3 predictive factors). In contrast, presently LRLR cannot be recommended for patients in the high difficulty class (4 or 5 preoperative predictive factors) because LRLR has a longer operative time without any benefits in other surgical outcomes for such patients, compared with ORLR.

## DISCLOSURES

Conflict of Interest: None.

Funding Information: This work was supported by the Health, Labour and Welfare Policy Research Grants from the Ministry of Health, Labour and Welfare of Japan (Policy Research for Hepatitis Measures [H30‐Kansei‐Shitei‐003]).

Author Contributions: Study design: Masahiko Kinoshita, Akishige Kanazawa, and Shoji Kubo designed the study. Acquisition of data: Masahiko Kinoshita, Akishige Kanazawa, Shogo Tanaka, Shigekazu Takemura, Ryosuke Amano, Kenjiro Kimura, Hiroji Shinkawa, Go Ohira, Kohei Nishio. Data analysis: Masahiko Kinoshita, Akishige Kanazawa, and Shoji Kubo. Manuscript drafted by Masahiko Kinoshita, Akishige Kanazawa, and Shoji Kubo. All the authors reviewed the manuscript.

## Supporting information

Table S1

Table S2
