# Supplementary material for: Indications of Laparoscopic Repeat Liver Resection for Recurrent Hepatocellular Carcinoma
Source: Ann Gastroenterol Surg. 2021 Aug 4;6(1):119–26. doi: 10.1002/ags3.12493 (PMC8786700; doi:10.1002/ags3.12493)
Supplement: Supplementary file 1 — Table S1 [file AGS3-6-119-s002.docx]

| Supplementary table 1. Comparison of preoperative backgrounds in low or intermediate difficulty class between patients who underwent laparoscopic and open repeat liver resection. | | | | | | | |
| --- | --- | --- | --- | --- | --- | --- | --- |
| Variables | | |  | | LRLR (n = 92) | ORLR (n = 44) | *P*-value |
|  |  | Age, median (range), years | | | 71 (32–86) | 68 (50–87) | 0.49 |
|  |  | Sex, male/female | | | 76/16 | 36/8 | 0.91 |
|  |  | Body mass index, median (range), kg/m^2^ | | | 23 (17–37) | 24 (17–31) | 0.52 |
|  |  | Comorbid liver disease, n (%) | | |  |  |  |
|  |  | Anti-HCV positive | | | 44 (48) | 26 (59) | 0.22 |
|  |  | HBs antigen positive | | | 26 (28) | 6 (14) | 0.060 |
|  |  | Alcoholic hepatitis | | | 7 (7.6) | 2 (4.6) | 0.49 |
|  |  | Non-alcoholic steatohepatitis | | | 2 (2.1) | 2 (4.6) | 0.59 |
|  |  | Tumor diameter, median (range), cm | | | 1.5 (0.4–3.8) | 1.7 (0.6–8.5) | 0.13 |
|  |  | A history of previous open liver resection, n (%) | |  | 40 (43) | 36 (82) | <0.0001 |
|  |  | A history of two or more previous liver resections, n (%) | |  | 15 (16) | 10 (23) | 0.37 |
|  |  | A history of previous major liver resection (not less than sectionectomy), n(%) | |  | 14 (15) | 6 (14) | 0.81 |
|  |  | Tumor near the resected site of the previous liver resection, n (%) | |  | 50 (54) | 24 (55) | 0.98 |
|  |  | Intermediate or high difficulty in the difficulty scoring system*, n (%) | |  | 37 (40) | 18 (41) | 0.94 |

LRLR, laparoscopic repeat liver resection; ORLR, open repeat liver resection; HCV, hepatitis C virus; HBs, hepatitis B surface.

*According to the difficulty scoring system^13^
