# Supplementary material for: Indications of Laparoscopic Repeat Liver Resection for Recurrent Hepatocellular Carcinoma
Source: Ann Gastroenterol Surg. 2021 Aug 4;6(1):119–26. doi: 10.1002/ags3.12493 (PMC8786700; doi:10.1002/ags3.12493)
Supplement: Supplementary file 2 — Table S2 [file AGS3-6-119-s001.docx]

| Supplementary table 2. Comparison of preoperative backgrounds in high difficulty class between patients who underwent laparoscopic and open repeat liver resection. | | | | | | | |
| --- | --- | --- | --- | --- | --- | --- | --- |
| Variables | | |  | | LRLR (n = 9) | ORLR (n = 15) | P-value |
|  |  | Age, median (range), years | | | 72 (46–79) | 65 (45–85) | 0.10 |
|  |  | Sex, male/female | | | 8/1 | 14/1 | 0.99 |
|  |  | Body mass index, median (range), kg/m^2^ | | | 25 (17–39) | 23 (17–30) | 0.21 |
|  |  | Comorbid liver disease, n (%) | | |  |  |  |
|  |  | Anti-HCV positive | | | 2 (22) | 4 (27) | 0.99 |
|  |  | HBs antigen positive | | | 3 (33) | 7 (42) | 0.68 |
|  |  | Alcoholic hepatitis | | | 1 (11) | 1 (6.7) | 0.99 |
|  |  | Non-alcoholic steatohepatitis | | | 0 (0) | 1 (6.7) | 0.99 |
|  |  | Tumor diameter, median (range), cm | | | 1.6 (0.9–3.0) | 1.6 (0.8–5.4) | 0.76 |
|  |  | A history of previous open liver resection, n (%) | |  | 9 (100) | 15 (100) | - |
|  |  | A history of two or more previous liver resections, n (%) | |  | 8 (89) | 4 (27) | 0.0094 |
|  |  | A history of previous major liver resection (not less than sectionectomy), n(%) | |  | 6 (67) | 13 (87) | 0.33 |
|  |  | Tumor near the resected site of the previous liver resection, n (%) | |  | 9 (100) | 15 (100) | - |
|  |  | Intermediate or high difficulty in the difficulty scoring system*, n (%) | |  | 6 (67) | 14 (93) | 0.13 |

LRLR, laparoscopic repeat liver resection; ORLR, open repeat liver resection; HCV, hepatitis C virus; HBs, hepatitis B surface.

*According to the difficulty scoring system^13^
